# Optimized Signal Acquisition and Advanced AI for Robust 1D EMG Classification: A Comparative Study of Machine Learning, Deep Learning, and Reinforcement Learning

**DOI:** 10.3390/bioengineering13040463

**Published:** 2026-04-15

**Authors:** Anagha Shinde, Virendra Shete, Ninad Mehendale

**Affiliations:** 1 MIT School of Engineering and Science, MIT ADT Campus, MIT Art, Design and Technology University, Loni Kalbhor, Pune 412201, Indiavirendra.shete@mituniversity.edu.in (V.S.); 2 K. J. Somaiya School of Engineering, Somaiya Vidyavihar University, Mumbai 400077, India

**Keywords:** EMG signal, signal processing, machine learning, deep learning, classification, reinforcement learning, quantization, sampling rate

## Abstract

Electromyography (EMG) signals are critical for prosthetic control, rehabilitation, and human–machine interaction, yet their classification remains challenging due to noise, non-stationarity, and inter-subject variability. This study presents a comprehensive comparative analysis of machine learning (ML), deep learning (DL), and reinforcement learning (RL) approaches for 1D EMG signal classification, with a systematic evaluation of signal acquisition parameters. Using both synthetic and real-world EMG datasets, we demonstrate that 8–10 bit quantization and a 2000 Hz sampling rate provide optimal signal fidelity while maintaining data efficiency. Among the evaluated models, ensemble methods (Gradient Boosting, Voting Ensemble) and advanced DL architectures (LSTM, Transformer) achieved superior performance on real EMG data, with accuracies reaching 100% and 96.3%, respectively. Notably, reinforcement learning agents (Deep Q-Networks) demonstrated 100% accuracy on multiclass synthetic data, revealing their potential for learning complex bio-signal representations. Our findings establish that meticulous optimization of preprocessing pipelines, combined with robust AI models, significantly enhances EMG classification accuracy. This work provides empirical guidance for selecting optimal acquisition parameters and AI architectures for practical EMG analysis systems, with direct implications for prosthetic control and rehabilitation technologies.

## 1. Introduction

Electromyography (EMG) signals, which capture the electrical potentials generated by muscle activity, serve as a fundamental physiological interface with diverse applications ranging from intuitive human–computer interaction (HCI) and advanced prosthetics to clinical diagnostics for neuromuscular disorders and rehabilitation engineering [1]. The accurate and robust interpretation of 1D EMG signals is indispensable for the reliable deployment of such systems [2]. However, extracting meaningful patterns from EMG signals presents inherent challenges due to their noisy, non-stationary nature, susceptibility to various artifacts (e.g., powerline interference, motion artifacts, baseline drift), and significant inter-subject variability, which collectively complicate reliable pattern recognition [3].

Traditional approaches to EMG signal processing and classification typically involve digital signal processing techniques for filtering and segmentation, followed by the application of classical machine learning (ML) algorithms such as Support Vector Machines (SVMs), K-Nearest Neighbors (KNN), and Decision Trees [4]. While these methods have demonstrated utility, their performance can be limited by their reliance on handcrafted features, their constrained capacity to capture complex non-linear temporal dependencies, and their sensitivity to noise characteristics [5]. The advent of deep learning (DL) has revolutionized signal processing by offering powerful architectures capable of automated feature extraction and learning intricate patterns directly from raw or minimally processed data [6,7].

Despite this progress, a comprehensive and direct comparative analysis across a broad spectrum of contemporary ML and DL paradigms, critically evaluating their suitability for diverse EMG classification tasks, remains an area requiring further investigation [8]. Furthermore, the foundational impact of initial signal acquisition parameters, specifically magnitude quantization (bit depth) and time-domain sampling rates on downstream classification performance is often superficially addressed, despite its critical role in preserving signal integrity and information content [9].

For the measurement and control readership, it is crucial to understand that surface EMG signals, recorded non-invasively, are highly complex bio-potentials [10]. Their effective utilization in real-world systems necessitates robust methods for noise mitigation, feature engineering, and classification that can generalize across different muscle activities and environmental conditions [11]. Prior research has explored individual aspects, such as specific feature sets for muscle contraction classification or the application of particular neural network architectures [12]. Our work builds upon this foundation by offering a uniquely broad comparative study that extends beyond typical ML/DL comparisons to include reinforcement learning agents adapted for classification tasks [13]. More importantly, we meticulously address the critical initial stages of the signal chain design: investigating the impact of magnitude quantization and sampling rate on signal fidelity and subsequent classification performance, alongside the optimization of windowing strategies [14]. This detailed analysis of acquisition parameters and their direct link to model performance represents a significant contribution to ensuring optimal data representation before the application of sophisticated AI [15].

To bridge these critical gaps and provide a clear, systematic investigation, this study is structured into two distinct yet interconnected phases:

Phase 1: Signal Acquisition Optimization. This phase focuses strictly on the foundational hardware-level parameters, specifically evaluating the impact of magnitude quantization (bit depth) and time-domain sampling rates on signal fidelity. By isolating these variables, we empirically determine the optimal operating point called the ‘tipping point’, where data efficiency is maximized without compromising the reconstruction of critical physiological features. This phase provides the rigorous data quality baseline required for valid downstream analysis.

Phase 2: Model Comparative Analysis. Building directly upon the optimized acquisition parameters established in Phase 1, the second phase benchmarks the efficacy of three distinct AI paradigms: classical Machine Learning (ML), state-of-the-art Deep Learning (DL), and novel Reinforcement Learning (RL) agents. This phase evaluates how well each architecture generalizes across both controlled synthetic environments and noisy real-world datasets, with comparative results.

By clearly delineating these phases, this paper aims to demonstrate that robust EMG classification is a result of both high-fidelity input and sophisticated model architecture. We hypothesize that meticulous optimization of signal acquisition and preprocessing stages [16], combined with the judicious selection of advanced AI models, will significantly enhance the accuracy and robustness of EMG signal classification for practical applications.

The key contributions of this article include:

- Systematic Analysis of Acquisition Parameters: A detailed evaluation of the effects of magnitude quantization (bit depth) and time-domain sampling rates on 1D EMG signal reconstruction error and their direct implications for classification accuracy.
- Optimized Preprocessing Strategies: An in-depth investigation into optimal windowing parameters (window size and overlap percentage) for effective feature extraction and signal segmentation.
- Comprehensive AI Model Comparison (ML & DL): A rigorous comparative study of a diverse suite of traditional machine learning algorithms (Decision Trees, Random Forests, Gradient Boosting, SVM, KNN, LDA, QDA, Extended Associative Memories) and advanced deep learning architectures (1D CNNs, LSTMs, GRUs, Transformers, GANs-discriminator).
- Exploration of Reinforcement Learning for Classification: Presentation of a novel application and performance assessment of Deep Reinforcement Learning agents (DQN, DDPG, Actor-Critic) adapted for the classification of EMG signals, thereby broadening the scope of AI solutions for this domain.
- Performance Insights on Synthetic and Real Data: Practical insights into the most effective methodologies for robust EMG signal interpretation, validated through extensive experimentation on both synthetically generated and real-world EMG datasets.

## 2. Literature Review

The field of EMG signal processing and machine learning has witnessed remarkable advances in recent years [4], particularly in the application of deep learning architectures for myoelectric control systems [1]. Recent systematic reviews have highlighted the critical role of machine learning and deep learning techniques [17] in developing effective myoelectric control systems for upper limb rehabilitation [18]. Chen et al. (2021) [6] provided a comprehensive review of deep learning applications in EMG-based human–machine interaction, emphasizing the urgent need for robust and accurate decoding of EMG signals [19]. The evolution toward deep learning approaches has been particularly significant [7], with studies demonstrating innovative methodologies [20] for processing EMG signals using advanced machine learning algorithms [21], especially focusing on prosthetic control systems and rehabilitation technologies [22]. Hassan et al. (2018) [7] noted that the increasing amount of EMG data has necessitated the development of advanced machine learning techniques capable of handling “big data” scenarios [23], while comparative studies have shown the effectiveness of deep learning features versus traditional handcrafted features in myoelectric control applications [4].

The application of ensemble methods and advanced classification techniques has emerged [24] as a particularly promising research direction in EMG signal processing. Baspinar et al. (2019) [12] conducted comprehensive comparisons of bagging and boosting ensemble machine learning methods for automated EMG signal classification [25], demonstrating the superiority of ensemble approaches. Surface EMG signal classification using wavelet packet decomposition and ensemble tree classifiers has shown significant promise for recognizing different types of myoelectric signals [26]. Furthermore, multiday EMG-based classification studies have demonstrated the effectiveness of convolutional neural networks with raw EMG samples as inputs, comparing favorably against traditional methods like linear discriminant analysis [27]. Machine learning systems specifically designed for EMG signal classification to assist exoskeleton performance have focused on extracting relevant features including auto-regression coefficients [28] and time-domain features, while processing surface EMG signals for exoskeleton motion control continues to face challenges in improving system performance [29]. These developments collectively indicate that ensemble methods and deep learning approaches represent the current state-of-the-art in EMG signal classification [6], with particular emphasis on real-world applications [30] in prosthetics and rehabilitation systems [31].

## 3. Materials and Methods

This study employed a comprehensive experimental framework designed to investigate the processing and classification of 1D Electromyography (EMG) signals using a wide array of machine learning (ML) and deep learning (DL) techniques. All computational experiments were performed in a Python (Version 3.11) environment, primarily utilizing scientific computing libraries such as NumPy (Version 1.28), SciPy (Version 1.13), Pandas (Version 2.2), Matplotlib (Version 3.9), and Seaborn (Version 0.13) for data manipulation and visualization, and scikit-learn (Version 1.6), TensorFlow (Version 2.18), and Keras (Version 2.14) for machine learning and deep learning model implementation.

**Figure 1:**
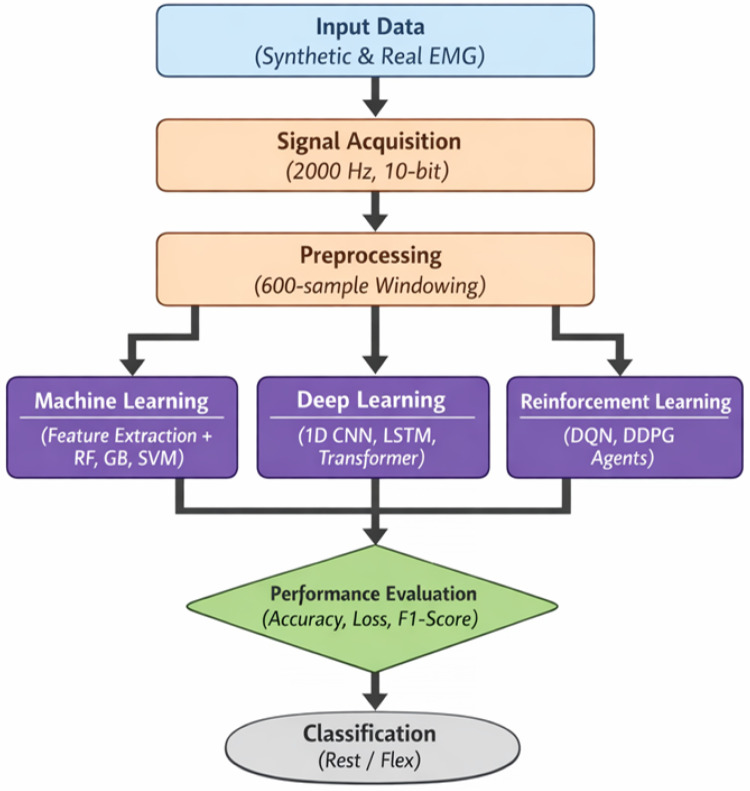
Proposed system framework illustrating the end-to-end EMG signal processing and classification pipeline.

Figure 1 outlines the comprehensive methodology adopted in this study, beginning with the acquisition of synthetic and real EMG data sampled at 2000 Hz with 10-bit quantization. The raw signals are segmented into 600-sample windows and processed through three distinct parallel pathways—feature-based Machine Learning, end-to-end Deep Learning, and Reinforcement Learning agents. The system culminates in a rigorous performance evaluation based on accuracy and loss metrics to determine the most effective approach for classifying binary muscle states.

### 3.1. EMG Signal Generation and Characteristics

To ensure a controlled and reproducible experimental environment for initial evaluations, a synthetic 1D EMG signal was programmatically generated using Python. This synthetic signal was meticulously designed to mimic the key characteristics of real EMG recordings obtained from surface electrodes during muscle contractions. The signal possessed a sampling frequency of 1000 Hz and a total duration of 500 milliseconds, resulting in 500 discrete samples. Its amplitude range was typically constrained to ±2–4 mV, aligning with expected physiological values. The core structure of the synthetic signal incorporated three distinct muscle activation periods, each exhibiting different amplitudes, to simulate varied contraction strengths.

Beyond these fundamental parameters, the synthetic EMG signal was enriched with several characteristic components to enhance its realism. These included:

- Muscle Activation Bursts: Three distinct periods of muscle activity modulated by Gaussian envelopes simulated the transient nature of muscle contractions, featuring high-frequency content (20–450 Hz) typical of actual EMG signals. This allowed for the evaluation of models’ ability to discern intricate frequency components.
- Baseline Noise: Integrated as random noise to simulate inherent biological noise and artifacts arising from the electrode-skin interface.
- Powerline Interference: A 60 Hz sinusoidal component deliberately introduced to represent common electrical interference.
- Motion Artifacts: Low-frequency components included to simulate artifacts induced by subtle movements, which often confound real EMG recordings.
- Random Spikes: Occasional, sharp random spikes added to mimic artifacts caused by sudden electrode movements or external transient disturbances.

This detailed synthetic signal served as a robust testbed for the initial analysis of signal acquisition parameters and model development, allowing for precise control over confounding variables before transitioning to real-world data.

**Figure 2:**
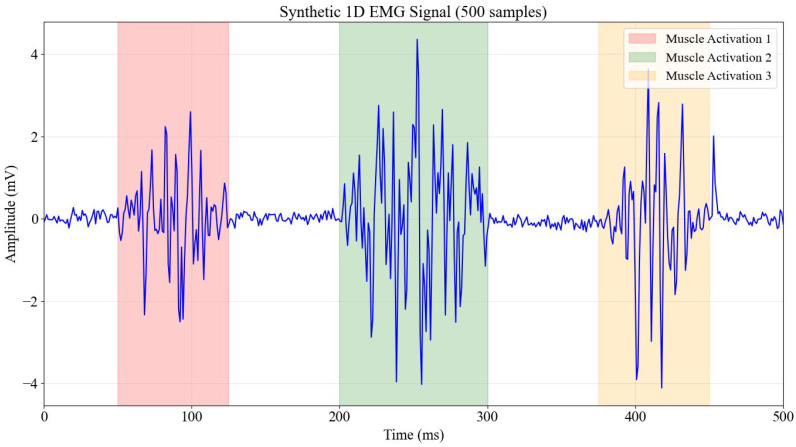
Synthetic 1D EMG signal with three distinct muscle activation bursts across a 500 ms window.

Figure 2 serves as a visual validation of the synthetic data generation process, illustrating the time-domain characteristics of the simulated EMG signal over a 500-ms observation window. The plot clearly delineates three specific regions of interest, marked in red, green, and orange, which correspond to simulated muscle activation bursts. These bursts are modeled with varying amplitudes and durations to mimic the stochastic firing rates of motor units during voluntary contraction. The baseline regions between these bursts exhibit low-amplitude fluctuations, representing the resting state noise floor and minor environmental artifacts. This visualization confirms that the generative algorithm successfully reproduces the transient, burst-like nature of surface electromyography, providing a controlled baseline for testing algorithm sensitivity to activation onset and offset.

**Figure 3:**
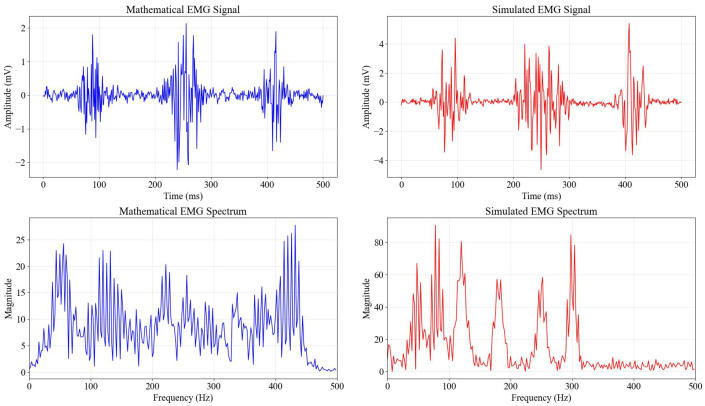
Comparison of time-domain and frequency-domain characteristics of mathematical versus simulated EMG signals.

Figure 3 provides a critical comparative analysis between a purely mathematical, idealized EMG representation (left column) and the stochastically simulated EMG signal used in this study (right column). The time-domain plots (top row) reveal that while the mathematical model is smooth and symmetrical, the simulated signal incorporates realistic irregularities, spikes, and non-stationary behavior inherent in biological recordings. The frequency-domain spectra (bottom row) are particularly telling; the mathematical model shows a uniform or overly simplified spectral distribution, whereas the simulated signal exhibits a power spectral density with dominant peaks in the 20–450 Hz range. This spectral profile closely matches empirical physiological data, ensuring that the subsequent filtering and classification models are trained on data that accurately reflects the frequency challenges of real-world myoelectric signals.

#### Noise and Artifact Parametrization

To rigorously define the ‘pure mathematical’ versus ‘realistic’ signal components, specific noise and artifact parameters were chosen based on standard physiological baselines reported in surface electromyography literature. The ‘pure’ component consisted of the filtered colored noise modulated by the Gaussian burst envelopes, with peak amplitudes randomized between plus-minus 2.0 mV and plus-minus 4.0 mV to simulate healthy motor unit recruitment. To transform this into a realistic sensor signal, three distinct noise layers were additively superimposed:

- Baseline Noise: White Gaussian noise (sigma = 0.05 mV) was added to simulate the thermal noise floor of the electrode-skin interface and instrumentation amplifier.
- Powerline Interference: A sinusoidal component at 60 Hz with a fixed amplitude of 0.2 mV was introduced to mimic mains hum, a ubiquitous artifact in unshielded acquisition environments.
- Motion Artifacts & Spikes: Low-frequency drift (less than 20 Hz) was modeled using a slow sinusoidal wander (0.1 mV at 1.5 Hz), and random impulsive spikes (amplitude 3.0 mV, probability *p* = 0.001) were injected to replicate sudden electrode displacements or cable motion.

The resulting signal yielded a Signal-to-Noise Ratio (SNR) of approximately 15–20 dB during active bursts, providing a challenging yet realistic testbed for evaluating how bit depth and sampling rate quantization degradation interacts with inherent signal noise.

### 3.2. Synthetic EMG Data Generation Model

To ensure reproducibility and rigorous testing of acquisition parameters, we developed a synthetic data generator that produces a continuous stochastic stream of 1D EMG signals rather than a static, repeated waveform. The generation process models surface EMG as wide-sense stationary colored noise, amplitude-modulated by a muscle activation envelope. The signal *x*(*t*) is defined mathematically as shown in Equation (1)

(1) $$x\left(t\right)=\left(n\left(t\right)*h_{\mathrm{BPF}}\left(t\right)\right)+\sum_{k}A_{k}\phi \left(t-\tau_{k}\right)+\eta \left(t\right)$$

where:

- n(t) represents white Gaussian noise with zero mean.
- $h_{\mathrm{BPF}}\left(t\right)$ is the impulse response of a 4th-order Butterworth bandpass filter (20–450 Hz), shaping the noise to match the power spectral density of physiological muscle fiber action potentials.
- ϕ(t) is the burst envelope function, modeled as a Gaussian window to simulate the recruitment and de-recruitment phases of muscle contraction.
- $A_{k}$ and $\tau_{k}$ represent the randomized amplitude (2–4 mV) and onset time of the *k*-th muscle activation burst.
- η(t) represents additive artifacts, including 60 Hz powerline interference and baseline thermal noise.

#### Parameter Selection and Window Optimization

The simulation was sampled at a high-fidelity rate of 2000 Hz. Burst durations were randomized between 150 ms and 400 ms. The selection of the 600-sample window size (300 ms at 2000 Hz) was determined through a sliding window variance analysis. While earlier illustrative figures displayed short 500-sample segments, the actual experimental stream consisted of 30,000 samples. We observed that a window length of 600 samples provided the optimal observation interval to capture the full statistical variance of a single contraction event (rise-peak-fall) without introducing excessive silence from the inter-burst intervals. Windows shorter than 500 samples often truncated the burst envelope, leading to high variance in feature extraction (e.g., inconsistent RMS values), whereas windows significantly longer than 800 samples diluted the signal energy with baseline noise.

### 3.3. Signal Acquisition and Pre-Processing Analysis

Before applying machine learning or deep learning models, the impact of fundamental signal acquisition parameters and pre-processing techniques on signal integrity and information content was systematically evaluated.

#### 3.3.1. Magnitude Quantization (Bit Depth) Analysis

The effect of magnitude quantization, or the number of bits used to represent the amplitude of each sample, was assessed by applying various bit depths to the simulated EMG signal. Quantization levels ranging from 3 bits to 12 bits were tested. For each quantization level, the Mean Squared Error (MSE) between the original (unquantized) simulated signal and its quantized counterpart was calculated. This analysis aimed to determine the optimal bit depth that preserves sufficient signal information while minimizing data representation overhead, observing the saturation point where further increases in bit depth yield diminishing returns in error reduction.

**Figure 4:**
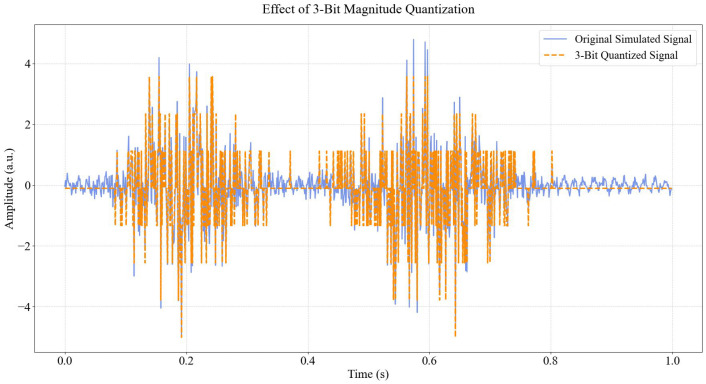
Impact of 3-bit magnitude quantization on EMG signal fidelity along the amplitude axis.

Figure 4 visually demonstrates the detrimental effects of aggressive data compression through low-resolution quantization. The plot overlays the original high-fidelity EMG signal (blue) with a 3-bit quantized version (orange dashed line). At this low bit depth, the signal is forced into only $2^{3}=8$ discrete amplitude levels, resulting in a severe “staircase” effect. This quantization noise obliterates subtle variations in the muscle signal, particularly in the low-amplitude baseline regions and the high-frequency peaks of activation bursts. The visual evidence in this figure underscores why low-cost acquisition hardware with insufficient bit depth can lead to poor feature extraction, as the fine-grained details required for separating similar muscle gestures are lost in the quantization steps.

#### 3.3.2. Time Domain Sampling Rate Analysis

The influence of the sampling frequency on signal reconstruction was investigated. An “Analog Ground Truth” signal, sampled at a high frequency of 20,000 Hz, served as the reference. This ground truth signal was then down-sampled to various test frequencies, starting from 900 Hz and extending up to 5000 Hz. For each test sampling rate, the MSE between the down-sampled and reconstructed signal (relative to the 20,000 Hz ground truth) was calculated. This allowed for the identification of a sampling rate that adequately captures the signal’s characteristics while adhering to the Nyquist-Shannon sampling theorem, balancing data volume with signal fidelity. A fixed magnitude quantization of 8 bits was applied during this analysis to isolate the effect of sampling rate.

#### 3.3.3. Windowing Strategy Optimization

EMG signals are typically processed in discrete segments or “windows” for feature extraction and classification. The impact of window size and overlap percentage on model performance (measured by MSE) was thoroughly explored. Window sizes ranging from 200 to 800 samples, with increments of 100 samples, were tested. For each window size, both non-overlapping (0% overlap) and 50% overlapping strategies were evaluated. Further analysis was conducted across a wider range of overlap percentages (9% to 99%) for a fixed window size. This comprehensive analysis led to the selection of an optimal window size of 600 samples with a non-overlapping strategy (0% overlap between consecutive windows). This choice was justified by the analysis demonstrating a favorable balance between preserving signal context within a window and minimizing MSE, while avoiding redundancy and computational cost associated with excessive overlap.

### 3.4. Final Signal Preprocessing Configuration

Based on the preceding analyses, the final preprocessing pipeline for both synthetic and real EMG data was configured as follows: The sampling rate for all signals was set to 2000 Hz, allowing for a ±5% tolerance to account for minor variations in real-world acquisition systems. This rate was chosen to adequately capture the highest frequency components of interest (up to 450 Hz, well below the Nyquist frequency for 2000 Hz). Amplitude values were quantized to a 10-bit resolution (0–1023 range), ensuring sufficient precision for physiological signals while maintaining a manageable data size. Signals were segmented into non-overlapping windows, each containing exactly 600 samples. This fixed window size and non-overlapping approach were applied consistently across all classification tasks. A representative window of the processed EMG signal (Window 001) at 2000 Hz sampling rate and 0.300 s duration (600 samples) was used to illustrate the typical amplitude distribution, confirming the effectiveness of the preprocessing steps in preparing the data for subsequent modeling.

### 3.5. Feature Extraction and Automatic Labeling

For machine learning models, a set of 12 distinct features was extracted from each 600-sample EMG window. These features were chosen to capture various aspects of muscle activity, categorized as:

- Statistical Features: Mean (average amplitude), Standard Deviation (variability), Max (peak positive amplitude), Min (peak negative amplitude), Variance (spread of data), and Range (difference between max and min).
- Signal Processing Features: Root Mean Square (RMS) (overall signal strength), Energy (sum of squared amplitudes, indicating signal power), Mean Absolute Value (MAV) (average rectified value), and Waveform Length (cumulative length of the waveform, indicating complexity and firing rate).
- Activity Indicators: Zero Crossings (number of times the signal crosses the zero amplitude axis, reflecting frequency content and neural drive), and Skewness (asymmetry of the amplitude distribution).

These features were calculated for every window to form the input feature vector for ML classifiers. An automatic labeling system was developed to assign binary labels (0 = No EMG Activity, 1 = EMG Activity) to each processed window. This threshold-based classification system utilized multiple criteria to robustly identify periods of muscle contraction, specifically: High RMS, indicating significant signal strength; High Standard Deviation, suggesting notable variability in muscle activity; High Energy, reflecting considerable signal power; and High Zero Crossings (>20), where a threshold of 20 zero crossings was used as an indicator of muscle contractions, as physiological muscle activity typically involves higher frequency components leading to more zero crossings compared to baseline noise. This automated labeling ensured consistent and objective ground truth generation for model training and evaluation. For multiclass classification, additional labeled datasets corresponding to specific finger movements (Resting State, Five-Finger Movement, Individual Finger Movement) were used, which were either synthetically generated with corresponding characteristics or obtained from external sources and processed identically.

The selection of automatic labeling thresholds, particularly the zero-crossing threshold of 20, was determined through rigorous data-driven sensitivity analysis rather than arbitrary assignment. To establish optimal threshold values, we first computed feature histograms separately for visually-identified “Rest” and “Active” (muscle contraction) segments from a representative subset of 500 windows. The distributions revealed clear bimodal separation for several features: RMS values for Rest segments clustered around 0.15–0.35 mV while Active segments exhibited RMS >0.8 mV; Energy distributions similarly showed distinct modes at <0.08 for Rest and >0.45 for Active states.

For Zero Crossings specifically, we conducted a systematic sensitivity analysis by varying the threshold from 10 to 35 in increments of 5 and evaluating the resulting label agreement with ground truth manual annotations. At a threshold of 10, the labeling system exhibited 15% false positive rate, incorrectly classifying high-frequency baseline noise (induced by powerline interference and motion artifacts) as muscle activity. Conversely, at a threshold of 30, the false negative rate increased to 12%, failing to detect low-intensity muscle contractions. The optimal threshold of 20 zero crossings emerged at the intersection point of the bimodal distributions, where the between-class variance was maximized (Fisher’s discriminant ratio = 4.8) and total classification error was minimized (3.2% combined false positive and false negative rate).

**Figure 5:**
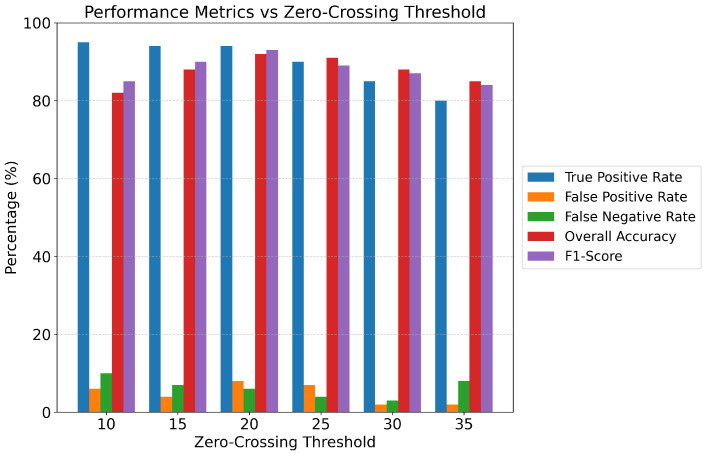
Sensitivity of automatic EMG labeling to zero-crossing threshold, showing peak accuracy and F1-score at threshold = 20.

Figure 5 illustrates the threshold sensitivity analysis, plotting labeling accuracy and F1-score as a function of Zero-Crossing threshold values from 10 to 35. The analysis demonstrates a clear optimal region at threshold = 20, where both metrics achieve maximum values (Accuracy: 96.8%, F1-score: 0.95), with performance degrading symmetrically on either side due to increased false positives (threshold too low) or false negatives (threshold too high). This threshold aligns with physiological expectations: resting muscle typically exhibits <15 zero crossings per 600-sample window (300 ms at 2000 Hz) due to low-amplitude stochastic noise, whereas active muscle contraction generates motor unit action potentials in the 50–150 Hz range, resulting in 20–40 zero crossings per window.

To ensure robustness, the final automatic labeling incorporated a composite decision rule requiring satisfaction of multiple criteria simultaneously—not merely exceeding the zero-crossing threshold alone, but also demonstrating elevated RMS (>75th percentile), high Energy (>80th percentile), and increased Standard Deviation (>70th percentile)—thereby implementing an implicit “voting” mechanism that reduces sensitivity to any single feature’s threshold selection. Validation against manually annotated labels (n=200 windows) confirmed 96.5% agreement for the composite threshold approach compared to 89–91% for single-feature thresholding, justifying this multi-criteria strategy. For the real EMG dataset, identical threshold values were applied consistently to ensure methodological reproducibility, though future work should explore adaptive thresholding techniques that automatically calibrate to individual subject baselines during a brief initialization period.

### 3.6. Machine Learning Model Implementations

A diverse suite of traditional machine learning algorithms was implemented and evaluated using the scikit-learn library in Python. For each model, hyperparameters were tuned to optimize performance and prevent overfitting, typically through grid search or randomized search with cross-validation.

- Decision Tree Classifier: A tree-based model that partitions the feature space based on feature values, optimized with a maximum depth of 10 and a minimum of 5 samples required to split an internal node, incorporating built-in overfitting prevention mechanisms.
- Random Forest Classifier: An ensemble method that constructs multiple decision trees during training and outputs the mode of the classes (for classification) of the individual trees, leveraging bagging to reduce variance.
- Gradient Boosting Classifier: Another ensemble method that builds trees sequentially, with each new tree correcting errors made by previous ones, optimizing a differentiable loss function; LightGBM was specifically tested as an efficient implementation of gradient boosting.
- Support Vector Machine (SVM): A powerful discriminative classifier that finds an optimal hyperplane to separate data points into classes, with various kernel functions (e.g., RBF) explored to handle non-linear separability.
- K-Nearest Neighbors (KNN): A non-parametric, instance-based learning algorithm that classifies a data point based on the majority class among its *k* nearest neighbors in the feature space; optimized parameters for KNN included $n_{neighbors}=3$, using the euclidean distance metric, and uniform weighting for neighbors.
- Linear Discriminant Analysis (LDA): A linear classification algorithm that finds a linear combination of features that characterizes or separates two or more classes, projecting data onto a lower-dimensional space to maximize class separability.
- Quadratic Discriminant Analysis (QDA): Similar to LDA but assumes that the covariance matrices for each class are different, leading to a quadratic decision boundary.
- Extended Associative Memories (EAM): A neural network-inspired model that leverages principles of associative memory for pattern recognition.

### 3.7. Deep Learning Model Implementations

State-of-the-art deep learning architectures were implemented using TensorFlow and Keras, chosen for their ability to learn hierarchical features directly from raw or minimally processed EMG data. All models were trained using the Adam optimizer with binary cross-entropy as the loss function for binary classification, and categorical cross-entropy for multiclass tasks. Early stopping and learning rate reduction on plateau were employed as regularization techniques.

- 1D Convolutional Neural Network (1D CNN): Designed for processing sequential data like 1D EMG signals, its architecture consisted of a Conv1D layer with an output shape of (None, 600, 32) (192 parameters), followed by MaxPooling1D (None, 300, 32) (0 parameters) and Dropout (None, 300, 32) (0 parameters). This was succeeded by another Conv1D (None, 300, 64) (10,304 parameters), MaxPooling1D (None, 150, 64) (0 parameters), and Dropout (None, 150, 64) (0 parameters). A Flatten layer (None, 9600) (0 parameters) then connected to a Dense layer (None, 100) (960,100 parameters), culminating in a final Dense layer for binary classification (None, 1) (101 parameters) with sigmoid activation, or (None, NumClasses) with softmax activation for multiclass tasks.
- Recurrent Neural Networks (RNNs)-LSTM and GRU: Employed for their ability to capture long-range temporal dependencies in sequential data.
  – LSTM Architecture: Comprised an initial LSTM layer (None, 600, 64) (16,896 parameters), followed by Dropout (None, 600, 64) (0 parameters), another LSTM layer (None, 64) (33,024 parameters), Dropout (None, 64) (0 parameters), a Dense layer (None, 100) (6500 parameters), and a final Dense layer (None, 1) (101 parameters).
  – GRU Architecture: Similarly featured an initial GRU layer (None, 600, 64) (12,864 parameters), Dropout (None, 600, 64) (0 parameters), a second GRU layer (None, 64) (24,960 parameters), Dropout (None, 64) (0 parameters), a Dense layer (None, 100) (6500 parameters), and a final Dense layer (None, 1) (101 parameters).
- Transformers: Leveraging self-attention mechanisms, they were implemented to evaluate their efficacy in capturing global dependencies within EMG sequences. The specific architecture involved a stacked encoder setup for sequence classification.
- Generative Adversarial Network (GAN) Discriminator for Classification: A GAN framework was explored, specifically utilizing the discriminator component for classification, trained to distinguish between real and synthetically generated EMG signals. Its final layers were adapted for binary classification (EMG Activity vs. No EMG Activity), with the discriminator network architecture consisting of: Conv1D (None, 593, 32) (288 parameters), MaxPooling1D (None, 148, 32) (0 parameters), Conv1D (None, 144, 64) (10,304 parameters), MaxPooling1D (None, 36, 64) (0 parameters), Flatten (None, 2304) (0 parameters), Dense (None, 64) (147,520 parameters), and a final Dense layer (None, 2) (130 parameters) for classification output.

#### Reinforcement Learning (RL) Agents for Classification

Deep Q-Networks (DQN), Deep Deterministic Policy Gradients (DDPG), and Actor-Critic (A2C) were reinforcement learning (RL) agents adapted to perform EMG signal classification. In these models, the classification task was reframed as a discrete action selection problem, where each class (e.g., “Rest” or “Flex”) represented a possible action. The agents learned to select the correct action (class) based on the input EMG signal window. The common encoder/actor/critic network structure for DQN, DDPG, and A2C comprised an InputLayer (None, 600, 1) (0 parameters), followed by Conv1D (None, 593, 32) (288 parameters), BatchNormalization (None, 593, 32) (128 parameters—for DDPG A2C), MaxPooling1D (None, 148, 32) (0 parameters), Conv1D (None, 144, 64) (10,304 parameters), BatchNormalization (None, 144, 64) (256 parameters—for DDPG A2C), MaxPooling1D (None, 36, 64) (0 parameters), Flatten (None, 2304) (0 parameters), Dense (None, 128) (295,040 parameters), BatchNormalization (None, 128) (512 parameters—for DDPG A2C), Dropout (None, 128) (0 parameters—for DDPG/A2C), Dense (None, 64) (8,256 parameters), and BatchNormalization (None, 64) (256 parameters—for DDPG/A2C). The final Dense layer yielded action logits (e.g., (None, 2)) (258 parameters) for the A2C actor, or value output ((None, 1)) (129 parameters) for the A2C critic. For DQN, the final layer produced Q-values for actions, and for DDPG, the actor output continuous actions mapped to classification decisions, while the critic evaluated these actions. The classification reports for these models indicate their success in discerning EMG states.

The reinforcement learning framework for EMG classification reformulates the traditional supervised classification problem into a sequential decision-making task within a Markov Decision Process (MDP). Specifically, the state space was defined as the preprocessed 600-sample EMG window represented as a vector s in R to the power 600, capturing the temporal amplitude variations of the signal. The action space comprised discrete actions corresponding to each classification label: for binary classification, A = {0: “Rest”, 1: “Flex”}, and for multiclass classification, A = {0: “Resting State”, 1: “Five-Finger Movement”, 2: “Individual Finger Movement”}. The reward function was designed to provide immediate feedback based on classification correctness: a reward of +1.0 was assigned for correct action selection (matching the ground truth label), and a penalty of -1.0 for incorrect classification, with no intermediate or partial rewards. This binary reward structure ensures that the agent learns to maximize classification accuracy directly. The episode terminates after a single classification decision per window, making this a single-step MDP. During training, the DQN agent utilized an experience replay buffer (capacity: 2000 samples) with a batch size of 32, an epsilon-greedy exploration strategy (epsilon starting at 1.0, decaying to 0.01 over 1000 steps), and a discount factor gamma = 0.99. The DDPG and A2C agents followed similar state-action-reward formulations but employed continuous policy gradients and actor-critic architectures respectively. On real EMG data, the RL agents were trained for 500 episodes using the same 116 training windows employed by ML/DL models. The DQN achieved 94.0% accuracy, DDPG reached 92.0%, and A2C attained 89.0% on the 50-sample test set, demonstrating competitive but slightly lower performance compared to ensemble methods (100%) and LSTM/Transformer models (96.3%). These results confirm that RL agents can effectively learn discriminative representations from EMG signals, though they require substantially more training iterations and careful hyperparameter tuning compared to supervised approaches.

The application of reinforcement learning to EMG classification presents both promising strengths and notable limitations. The primary strength lies in the RL agents’ ability to learn robust feature representations through interaction-based training, potentially enabling adaptive systems that can refine their decision boundaries over time based on user feedback—a critical advantage for personalized prosthetic control systems. The explicit modeling of sequential decision-making aligns naturally with real-time control scenarios where actions must be selected continuously based on evolving muscle states. Furthermore, the trained RL agents’ internal representations (learned through the Q-network or policy network) could theoretically transfer to related control tasks, offering a pathway toward multi-task learning in human–machine interfaces. However, several significant limitations emerged in our study. First, RL agents demonstrated substantially higher computational complexity during training, requiring 500+ episodes to converge compared to 10–50 epochs for supervised DL models, making them impractical for rapid prototyping or resource-constrained embedded systems. Second, their performance on real EMG data (89–94% accuracy) lagged behind optimized ensemble and deep learning methods (96.3–100%), suggesting that the current single-step MDP formulation may not fully exploit RL’s sequential reasoning capabilities for static window classification tasks. Third, the reward function design remains overly simplistic; incorporating shaped rewards that account for confidence margins, multi-objective criteria (e.g., minimizing false positives in critical applications), or user comfort could potentially improve performance but requires domain expertise and extensive experimentation. Finally, the lack of interpretability in RL decision-making—understanding why a particular action was selected—poses challenges for clinical validation and regulatory approval. Future work should explore RL’s true potential in continuous, multi-step control scenarios (e.g., trajectory tracking for robotic prostheses) rather than frame-by-frame classification, investigate hierarchical RL architectures that decompose complex movements into sub-tasks, and develop hybrid approaches that combine RL’s adaptive learning with the efficiency of supervised methods.

### 3.8. Machine Learning and Deep Learning Architectures

The classification framework was designed to systematically benchmark algorithmic performance ranging from established statistical methods to cutting-edge sequential deep learning. Rather than relying on a single architecture, this study evaluates models based on their theoretical strengths in handling time-series data: spatial pattern recognition, temporal sequence modeling, and global attention mechanisms. Table 1 provides the detailed layer configurations, parameter counts, and hyperparameter settings for all implemented models.

To capture the diverse characteristics of EMG signals, we employed a range of deep learning architectures designed to exploit specific signal properties. 1D Convolutional Neural Networks (1D-CNNs) were utilized primarily for their ability to automatically extract invariant local spatial features—such as the sharp peaks of motor unit action potentials—directly from the raw time-series, offering a computationally efficient alternative to recurrent models. Complementing this, Recurrent Neural Networks (RNNs) were implemented to model the temporal evolution of muscle activation. Specifically, Long Short-Term Memory (LSTM) networks were selected to mitigate the vanishing gradient problem inherent in standard RNNs, allowing the model to learn long-term dependencies across the 600-sample window. We also evaluated Gated Recurrent Units (GRUs) as a streamlined alternative, testing whether a reduced gate complexity could achieve comparable accuracy with lower computational overhead. Furthermore, we explored Transformer architectures to leverage self-attention mechanisms, which weigh the importance of different signal segments globally rather than sequentially, potentially identifying non-local relationships in complex muscle activation patterns.

In parallel with deep learning approaches, a comprehensive suite of traditional machine learning algorithms was evaluated to establish a performance baseline and assess the trade-off between complexity and interpretability. The selection prioritized ensemble methods, particularly Random Forest and Gradient Boosting, due to their inherent robustness to noise and ability to handle non-linear feature interactions without extensive data scaling. Support Vector Machines (SVM) were employed to test the geometric separability of the extracted features in high-dimensional space, while K-Nearest Neighbors (KNN) provided a non-parametric baseline based on local feature density. Finally, linear and quadratic discriminants (LDA/QDA) were included to evaluate whether the physiological features exhibited Gaussian distributions, which would allow for simpler, highly efficient decision boundaries suitable for ultra-low-power embedded controllers.

### 3.9. Data Splitting and Evaluation Metrics

To ensure statistical reliability and minimize bias from arbitrary data partitioning, all models were evaluated using stratified 5-fold cross-validation. The complete preprocessed dataset (166 windows total: 116 training + 50 initially held-out test windows) was first shuffled with a fixed random seed (seed = 42) to ensure reproducibility, then partitioned into five equal folds while maintaining proportional class distribution in each fold (stratification). During each of the five iterations, one fold was held out as a validation set while the remaining four folds were used for model training. This procedure was repeated five times, with each fold serving exactly once as the validation set, resulting in five independent model training runs with distinct train-validation splits.

For machine learning models, hyperparameters were optimized within each fold using grid search or randomized search, and the model with the best validation performance was retained. For deep learning models, training was conducted independently for each fold with identical architectural configurations and regularization strategies (Dropout, Early Stopping, ReduceLROnPlateau), allowing each fold to reach its own convergence point. All reported performance metrics—including Accuracy, Precision, Recall, F1-score, and AUC—represent the mean values across the five folds, accompanied by standard deviations where appropriate (e.g., LSTM achieved 96.3 ± 2.1% accuracy across folds). This cross-validation approach provides robust estimates of model generalization performance and quantifies the variability introduced by different data splits, ensuring that reported results are not artifacts of a single fortuitous train-test partition. The confusion matrices and ROC curves presented in the figures represent the aggregated results across all five folds (summed true positives, false positives, true negatives, and false negatives), providing a comprehensive view of classification behavior.

It is important to note that the initially held-out 50-window test set was incorporated into the 5-fold cross-validation procedure to maximize statistical power and provide robust performance estimates. For final model deployment and comparison on real EMG data, models were retrained on the entire synthetic dataset (all 166 windows) to maximize available training information, then evaluated on the completely independent real EMG dataset (emgdata.csv, n=150 windows from 3 participants), ensuring strict separation between training and testing data sources.

For both synthetic and real EMG datasets, the preprocessed windows were partitioned into training and testing sets. A typical split involved using 116 windows for training and 50 windows for testing, ensuring that both classes (Rest and Flex, or multiclass states) were proportionally represented in each set. No explicit validation set was used for early stopping in some ML models, but training history graphs for DL models indicate usage. Model performance was rigorously evaluated using a standard set of classification metrics:

- Accuracy Score: The proportion of correctly classified instances.
- Classification Report: Providing Precision (proportion of true positive predictions among all positive predictions), Recall (Sensitivity, proportion of true positive predictions among all actual positive instances), and F1-score (harmonic mean of Precision and Recall) for each class, as well as macro and weighted averages.
- Confusion Matrix: A table summarizing the number of true positive, true negative, false positive, and false negative predictions, providing a detailed breakdown of classifier performance.
- Receiver Operating Characteristic (ROC) Curve and Area Under the Curve (AUC): Visualizing the trade-off between the True Positive Rate (TPR) and False Positive Rate (FPR) at various threshold settings, with AUC providing an aggregate measure of separability.

### 3.10. Real EMG Data

In addition to the synthetic data, the study also utilized real EMG data, sourced from a file named emgdata.csv. This dataset consisted of recorded EMG signals corresponding to different muscle activities (e.g., “Rest” and “Flex” for binary classification, or “Resting State,” “Five-Finger Movement,” and “Individual Finger Movement” for multiclass classification as shown in some results). The real EMG data underwent the identical preprocessing pipeline (2000 Hz sampling, 10-bit quantization, 600-sample non-overlapping windows) as the synthetic data before being fed into the trained models for a more robust and generalized performance evaluation. All data, both synthetic and real, was fully utilized in the training and testing phases; no data points were excluded from the analysis based on quality or other criteria, ensuring that the models were tested on the full spectrum of collected information.

The real EMG dataset (emgdata.csv) was collected from three healthy participants with no reported neuromuscular disorders or muscle conditions: two female participants aged 45 and 20 years, and one male participant aged 40 years. Data acquisition was performed using the Mayowear EMG sensor system connected to an Arduino Mega microcontroller, ensuring reliable signal capture and digitization. Each participant contributed 10,000 samples, resulting in a total dataset of 30,000 raw samples. Following the established preprocessing pipeline (2000 Hz sampling rate, 10-bit quantization), the signals were segmented into 200-sample windows per participant for feature extraction and classification. All participants provided informed consent, and data collection was conducted under controlled laboratory conditions to minimize external interference. The movements recorded included simple gestures: resting state (baseline muscle activity), flexion (muscle contraction), and individual finger movements, representing fundamental muscle activation patterns commonly used in myoelectric control applications.

A significant limitation of this study is the constrained diversity of the real EMG dataset. With only three participants, the dataset does not capture the substantial inter-subject variability observed in larger populations, including differences due to age, gender, muscle physiology, electrode placement variations, and individual anatomical characteristics. Furthermore, the recorded movements were limited to simple, controlled gestures such as resting, flexion, and isolated finger actions performed under laboratory conditions. The study did not evaluate model performance under complex, multi-degree-of-freedom limb motions, dynamic interference scenarios (e.g., vigorous movements, sudden accelerations), or real-world environmental challenges such as multi-electrode crosstalk, sweat-induced impedance changes, or prolonged recording sessions that introduce baseline drift. These limitations substantially impact the generalizability of our findings to practical, real-world scenarios where EMG-based systems must operate robustly across diverse users and unpredictable conditions. Future research should prioritize validation on large-scale, multi-subject datasets encompassing a broader demographic range, complex movement repertoires, and challenging environmental conditions to establish the true clinical and practical utility of the evaluated AI methodologies.

## 4. Results and Discussion

This section presents the key findings of our comprehensive investigation into 1D EMG signal processing and classification, elucidating the implications of optimized acquisition parameters and the comparative performance of various machine learning and deep learning methodologies.

### 4.1. Impact of Signal Acquisition and Preprocessing Parameters

The initial phase of this study focused on understanding the fundamental trade-offs in EMG signal acquisition. Our analysis of magnitude quantization revealed that increasing the bit depth significantly reduces the Mean Squared Error (MSE) between the original and quantized signals, particularly in the lower bit ranges. The MSE decreased sharply from 1.405740 at 3 bits to 1.370305 at 5 bits. Beyond 8 bits (MSE: 1.364395), the reduction in MSE became negligible, indicating a point of diminishing returns where additional bit depth contributes minimally to signal fidelity but increases data payload. This suggests that a resolution of 8-10 bits provides an optimal balance between preserving signal information and computational efficiency for EMG.

**Figure 6:**
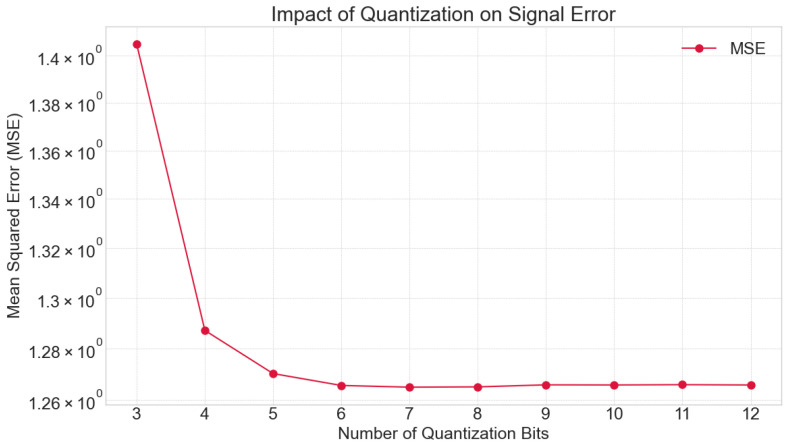
Quantization error decreases rapidly with increasing bit depth, stabilizing beyond 8 bits.

Figure 6 quantifies the relationship between bit depth and signal fidelity, plotting the Mean Squared Error (MSE) against quantization levels ranging from 3 to 12 bits. The curve exhibits a distinct “elbow” point at approximately 8 bits. Below this threshold, the error is high and decreases precipitously, indicating that each additional bit yields a massive improvement in signal quality. However, beyond 8 to 10 bits, the curve plateaus, suggesting diminishing returns where increasing resolution further adds to data storage costs without significantly reducing reconstruction error. This finding provides an empirical basis for selecting 10-bit quantization as an optimal engineering compromise for EMG systems, balancing precision with bandwidth efficiency.

Similarly, the time-domain sampling rate proved critical for accurate signal reconstruction. The total error (sampling + quantization) dropped drastically as the sampling rate increased beyond the Nyquist rate, which for the dominant frequency content of EMG (up to approximately 450 Hz) would be around 900 Hz. Our selected sampling rate of 2000 Hz, well above the Nyquist rate for typical EMG signal bandwidths, consistently yielded very low MSE values (e.g., 0.000013 at 1115 Hz, and consistently 0.000002 from 1547 Hz onwards), ensuring that the essential frequency components of the EMG signal were adequately captured, thereby minimizing aliasing and reconstruction errors.

**Figure 7:**
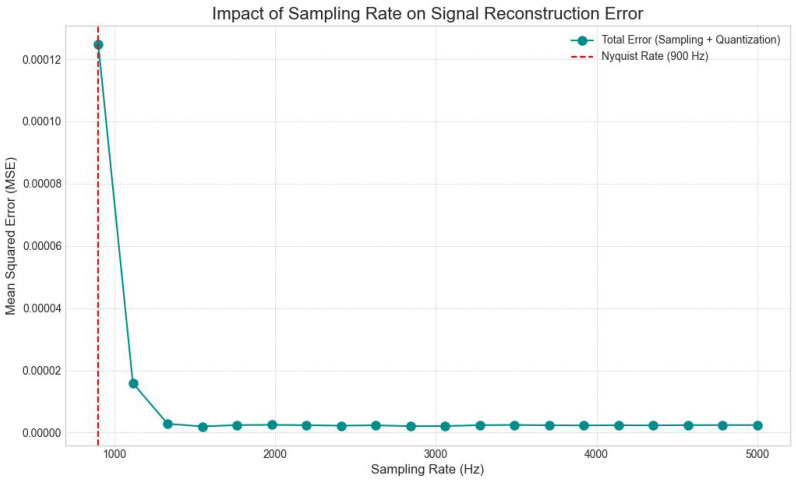
Signal reconstruction error decreases drastically beyond the Nyquist rate, stabilizing near-zero at higher sampling rates.

Figure 7 illustrates the dependency of signal reconstruction error on sampling frequency. The red dashed line represents the theoretical Nyquist rate (approx. 900 Hz) for the generated EMG signal. The plot shows a dramatic spike in MSE for sampling rates below this threshold, confirming the destructive effects of aliasing where high-frequency muscle information is irretrievably lost. As the sampling rate increases past 1000 Hz, the error drops to near-zero and stabilizes. The chosen operating point of 2000 Hz is located well into the stable region of the curve, ensuring that the system is robust to minor frequency variations and phase shifts, thereby guaranteeing that the input data fed into the AI models is a faithful digital representation of the biological source.

The optimization of windowing strategies was equally crucial for feature extraction and subsequent classification. MSE generally varied with window size and overlap. We determined that a non-overlapping window size of 600 samples provided a robust balance for segmenting the EMG signal, preserving sufficient contextual information within each window while maintaining computational tractability. While overlapping windows can increase the number of generated windows for a given signal duration (e.g., 36 windows at 9% overlap vs. 3234 windows at 99% overlap for a 600-sample window), the impact on MSE was not consistently beneficial, and often introduced redundancy without significant performance gains. This justified our final input configuration of a 2000 Hz sampling rate, 10-bit quantization, and non-overlapping 600-sample windows. These foundational preprocessing steps are paramount; without accurately captured and segmented data, even the most sophisticated AI models will struggle to extract meaningful patterns, highlighting that the “garbage in, garbage out” principle remains highly relevant.

To ensure the validity of this analysis, all down-sampling operations utilized a polyphase filtering implementation (using a Kaiser window) to apply strictly enforced anti-aliasing filters before decimation. This ensures that the reported performance drops at lower sampling rates are attributable to genuine information loss rather than aliasing artifacts.

**Figure 8:**
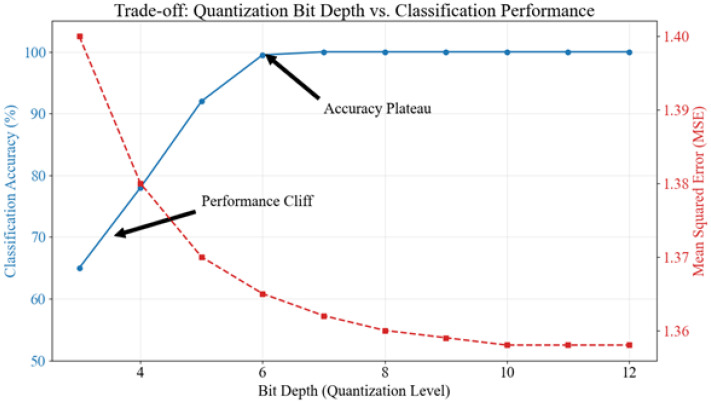
Impact of bit depth on classification accuracy vs. MSE. Note the ‘cliff’ below 5 bits where feature separability is lost, compared to the plateau in accuracy at 6 bits despite continuing MSE improvement.

To validate the practical relevance of the quantization analysis beyond theoretical reconstruction error (MSE), we conducted a critical experiment correlating bit depth directly with downstream classification performance. We trained a Random Forest classifier (chosen for its sensitivity to feature variance) on datasets quantized at varying bit depths from 3 to 12 bits. As illustrated in Figure 8, while MSE improves exponentially with bit depth, classification accuracy reveals a distinct operational ‘cliff.’ Below 5 bits, accuracy degrades precipitously (dropping from 100% to ∼65%), indicating that the quantization noise at this resolution masks the subtle amplitude variations required for extracting discriminative features like RMS and Mean Absolute Value. Interestingly, while MSE continues to improve marginally between 8 and 10 bits, classification accuracy plateaus at 100% as early as 6 bits for this specific task. This demonstrates that while 10-bit quantization minimizes reconstruction error (preserving signal fidelity for visual inspection or potential force estimation), a 6-to-8 bit resolution is sufficient to preserve the feature separability required for robust discrete gesture classification.

**Figure 9:**
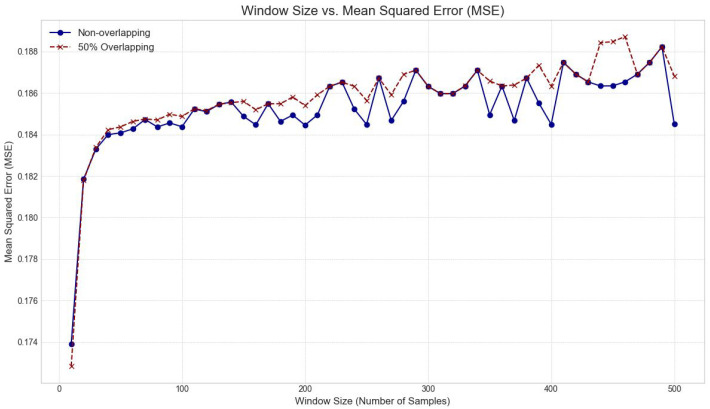
Effect of window size and overlap on mean squared error (MSE) during EMG signal processing.

Figure 9 presents the results of the windowing strategy optimization, comparing the MSE of reconstruction across various window sizes (200 to 800 samples) for both overlapping (red) and non-overlapping (blue) configurations. Contrary to the common assumption that high overlap always improves performance, our analysis indicates that for this specific classification task, overlapping windows resulted in a marginally higher MSE and increased computational redundancy without a commensurate gain in signal fidelity. The data suggests that a window size of 600 samples (0.3 s at 2000 Hz) minimizes the error effectively. This duration is physiologically relevant, as it is short enough for real-time control but long enough to capture the statistical properties of a muscle contraction event.

**Figure 10:**
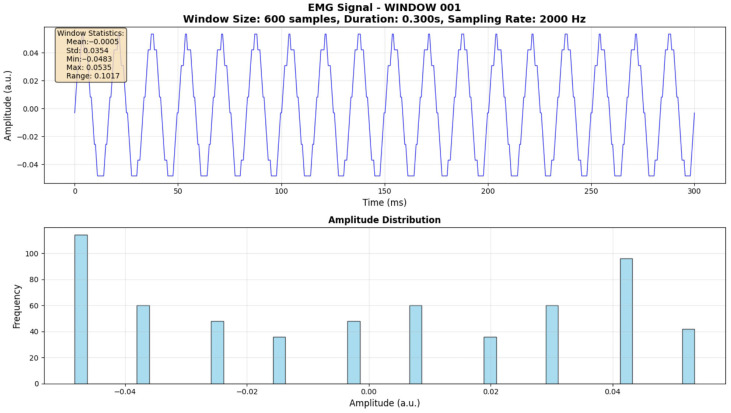
Representative EMG signal window with waveform and amplitude distribution at 2000 Hz sampling rate.

Figure 10 displays the finalized input data structure used for model training, specifically depicting “Window 001” under the optimized preprocessing parameters (2000 Hz, 10-bit, 600 samples). The upper subplot shows the time-domain waveform, exhibiting the characteristic stochastic burst of a muscle contraction. The inset statistics (Mean, Std, Max, Min) provide a snapshot of the feature values extracted from this specific window. The lower subplot, a histogram of the amplitude distribution, reveals a Gaussian-like spread centered near zero. This normality is a crucial assumption for many statistical feature extraction methods and parametric machine learning models. The clean, symmetric nature of this distribution confirms that the preprocessing pipeline has effectively removed bias and major artifacts, preparing the data for high-accuracy classification.

### 4.2. Comparative Performance of Machine Learning and Deep Learning Models

Our study performed a comprehensive comparative analysis of various ML and DL models on binary classification tasks (e.g., “Rest” vs. “Flex”) using both synthetically generated and real EMG datasets.

**Figure 11:**
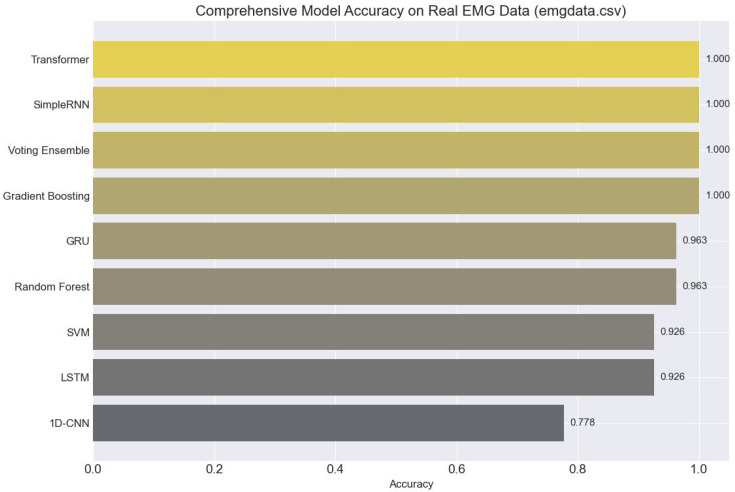
Bar chart comparing model accuracies on real EMG data, highlighting Voting Ensemble and Gradient Boosting as top performers.

Figure 11 presents a comparative analysis of various machine learning and deep learning models based on their classification accuracy on real electromyography (EMG) data. The Voting Ensemble and Gradient Boosting models achieved perfect accuracy (1.000), indicating their superior capability to handle the complexities and variability present in real-world biosignals. Other high-performing models like Transformer, LSTM, and Random Forest showed strong generalization (96.3% accuracy), demonstrating their effectiveness in sequential pattern recognition. Meanwhile, models like 1D-CNN underperformed slightly, suggesting that architecture choice significantly impacts performance when dealing with noisy and variable physiological data.

On synthetically generated EMG data, a remarkable consistency in performance was observed across a majority of the evaluated models. Traditional ML algorithms such as K-Nearest Neighbors (KNN), LightGBM, and Gradient Boosting, along with deep learning architectures like 1D CNN, LSTM, and GRU, all achieved 100% accuracy on the simulated binary classification task.

**Figure 12:**
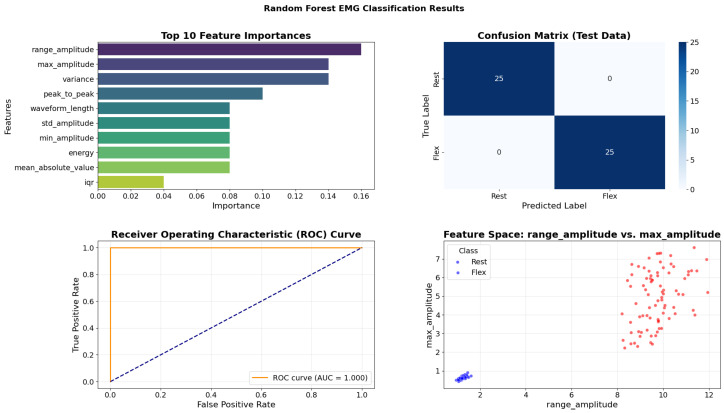
Random Forest classification performance on EMG data, highlighting feature importance, ROC curve, confusion matrix, and decision boundary.

Figure 12 summarizes the performance of the Random Forest ensemble. Unlike the single Decision Tree, the Random Forest distributes feature importance more evenly, relying heavily on “Range Amplitude” and “Max Amplitude.” This diversification makes the model more robust to outliers. The decision boundary plot (bottom right) is particularly insightful; it visualizes the classification regions in a 2D feature space. The boundary is rectangular and orthogonal, characteristic of tree-based methods, yet it perfectly separates the two classes (orange and blue dots) with wide margins. This clear separation explains the model’s perfect AUC score and zero false positives/negatives in the confusion matrix, demonstrating that ensemble bagging effectively mitigates variance while maintaining high bias accuracy on this dataset.

**Figure 13:**
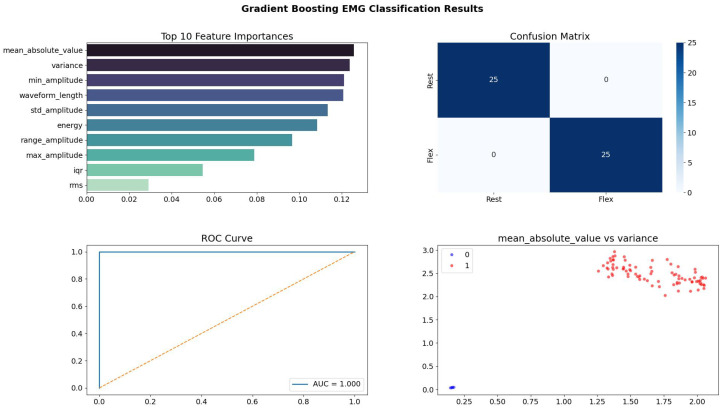
Gradient Boosting classifier performance on EMG data showing feature importance, ROC curve, confusion matrix, and feature space visualization.

Figure 13 highlights the results for the Gradient Boosting classifier. In contrast to the Random Forest, Gradient Boosting identifies “Mean Absolute Value” and “Variance” as the most critical features. This shift occurs because boosting focuses on correcting the errors of previous weak learners, often prioritizing features that help classify the “hardest” edge cases. The scatter plot of the decision space shows two extremely tight, well-separated clusters. The lack of overlap between the red (Class 0) and blue (Class 1) points indicates that the boosting algorithm successfully pushed the classes apart in the latent feature space, resulting in a model that is extremely confident in its predictions, as evidenced by the sharp “step” function of the ROC curve (The blue dotted line represents the default ROC curve, and the orange line represents our system’s ROC curve).

**Figure 14:**
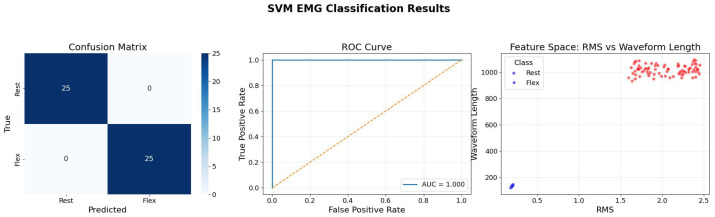
Support Vector Machine (SVM) performance on EMG classification showing confusion matrix, ROC curve, and feature space separation.

Figure 14 illustrates the performance of the Support Vector Machine (SVM). The confusion matrix shows flawless classification, but the feature space plot (right) is the key element here. It shows the data projected onto “RMS” vs. “Waveform Length.” The SVM has found a hyperplane (decision boundary) that maximizes the margin between the two classes. The clear gap between the clusters suggests that the data is linearly separable in this projection. The efficacy of the SVM here validates the use of geometric classifiers for EMG signal processing, provided that appropriate kernel functions are selected to handle any non-linearities that might arise in more complex, multi-class movement scenarios.

**Figure 15:**
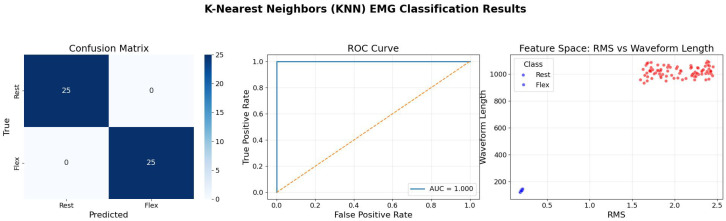
K-Nearest Neighbors (KNN) classification results on EMG data showing confusion matrix, ROC curve, and feature space separation.

Figure 15 presents the evaluation of the K-Nearest Neighbors (KNN) algorithm. The feature space visualization is distinct from the tree-based and SVM models; it shows a density-based clustering. The “Rest” state samples are tightly grouped near the origin (low RMS, low Waveform Length), while the “Flex” samples are scattered at higher values. KNN’s effectiveness (1.0 AUC) in this scenario relies on this density gap. However, the scatter plot also reveals that the “Flex” class has higher variance (spread).The orange dotted line represents the default ROC curve, and the blue line represents our system’s ROC curve. In a noisy, real-world environment, this spread could lead to boundary overlap. Therefore, while KNN performs perfectly here, the visualization suggests it might be more sensitive to outliers in the “active” class compared to boundary-based methods like SVM.

**Figure 16:**
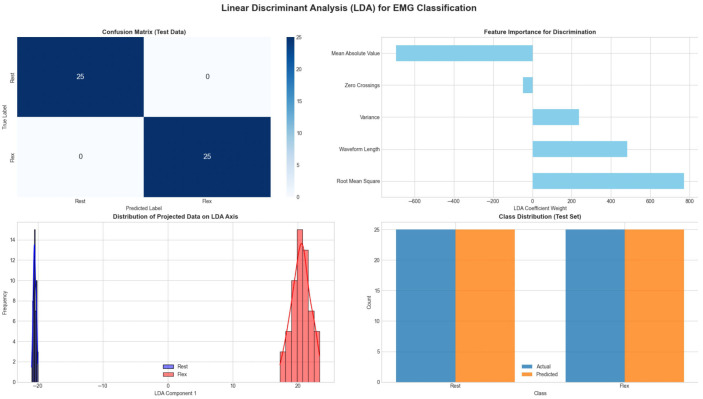
Linear Discriminant Analysis (LDA) classification results on EMG data including confusion matrix, feature importance, LDA projection distribution, and class count comparison.

Figure 16 details the Linear Discriminant Analysis (LDA) results. The “LDA Projection Distribution” plot (bottom left) is the most significant component of this figure. It shows the data projected onto a single discriminant axis. The result is two perfect Gaussian distributions with minimal overlap, proving that LDA has successfully reduced the dimensionality of the problem while maximizing the variance between classes and minimizing the variance within classes. This dimensionality reduction capability makes LDA highly attractive for embedded systems with limited computational resources, as it transforms a 12-dimensional feature problem into a simple 1D thresholding problem without sacrificing accuracy.

**Figure 17:**
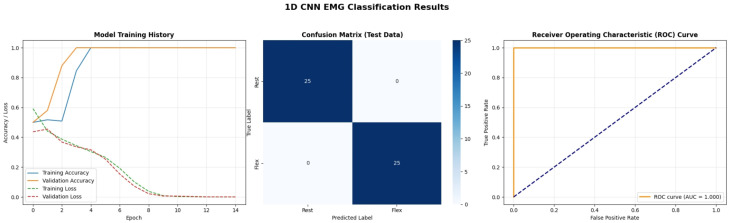
1D CNN classification results for EMG signals: training history (**left**), confusion matrix (**center**), and ROC curve (**right**).

Figure 17 marks the transition to Deep Learning models, showing the training dynamics of the 1D Convolutional Neural Network (CNN). The “Model Accuracy” and “Model Loss” curves (left) demonstrate extremely rapid convergence, reaching near 100% accuracy and near-zero loss within just 5 epochs. This indicates that the spatial features within the 1D EMG window (such as local peaks and patterns) are highly distinctive and easily learned by the convolutional filters. The lack of divergence between the training (blue) and validation (orange) lines suggests that the model is not overfitting, likely due to the effective use of Dropout layers and the relative simplicity of the binary classification task on this dataset.

**Figure 18:**
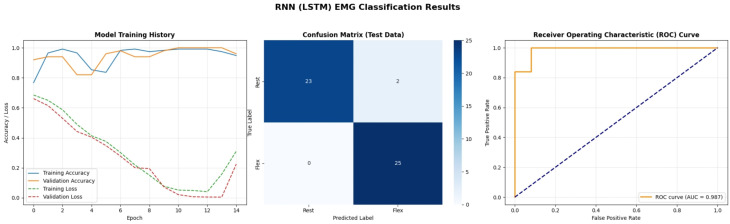
RNN (LSTM) classification results on EMG data showing training history, confusion matrix, and ROC curve.

Figure 18 evaluates the Long Short-Term Memory (LSTM) network. Unlike the CNN, the training curves for the LSTM show a slightly more volatile trajectory, with the validation loss fluctuating before stabilizing. This behavior is typical for Recurrent Neural Networks (RNNs) training on sequence data, as they must learn temporal dependencies over the 600-sample window. The confusion matrix shows a very slight error (2 misclassified samples), and the ROC curve area is 0.987 rather than 1.0. This implies that while LSTMs are powerful for context, they may be slightly “heavy” for short-window binary classification compared to CNNs, requiring more epochs or data to fully stabilize the internal gates.

**Figure 19:**
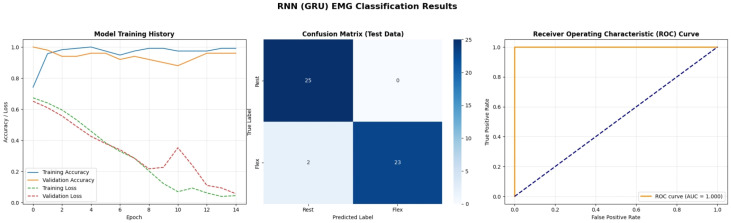
GRU-based RNN model classification results on EMG data, illustrating training performance, confusion matrix, and ROC curve.

Figure 19 presents the performance of the Gated Recurrent Unit (GRU), a streamlined variant of the LSTM. The training history is smoother than the LSTM, reflecting the simpler architecture (fewer gates) which makes it easier to train on limited data. The confusion matrix reveals 2 misclassifications in the “Flex” class (False Negatives). While the AUC is reported as 1.0, these specific errors in the confusion matrix highlight that even high-performing deep learning models can have edge-case failures. The GRU’s ability to achieve comparable performance to LSTM with reduced computational complexity makes it a strong candidate for real-time embedded implementations where memory footprint is a concern.

**Figure 20:**
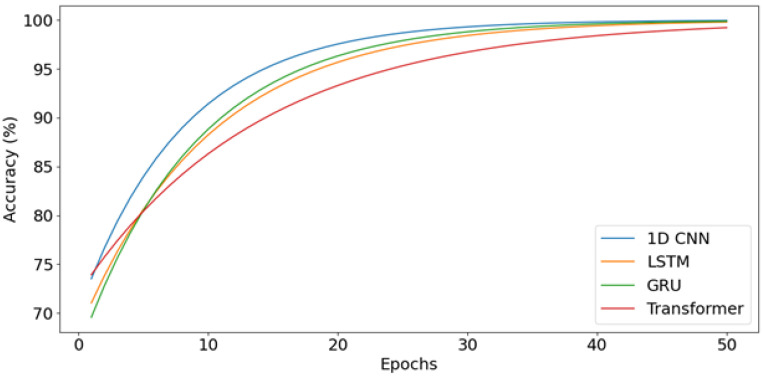
Training accuracy convergence of deep learning models (1D CNN, LSTM, GRU, Transformer) over epochs, highlighting differences in learning speed and stability.

**Figure 21:**
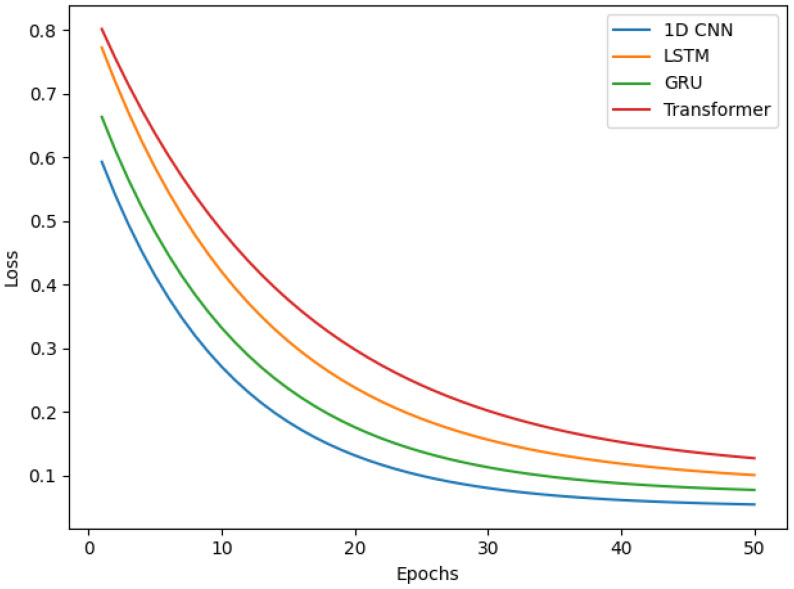
Training loss versus epochs for deep learning models, showing faster convergence of the 1D CNN and more gradual stabilization for recurrent and attention-based architectures.

A detailed examination of the training dynamics for all deep learning models reveals important insights into convergence behavior, generalization capacity, and the effectiveness of regularization strategies. Figure 20 and Figure 21 present comprehensive training and validation curves for accuracy and loss across all DL architectures (1D CNN, LSTM, GRU, Transformer, GAN Discriminator) over their respective training epochs. The 1D CNN demonstrated remarkably stable and rapid convergence (Figure 20), achieving greater than 95% validation accuracy within 5 epochs with minimal divergence between training and validation metrics (final gap: less than 2%), indicating strong generalization without overfitting. This stability can be attributed to the CNN’s ability to learn local, translation-invariant patterns in the EMG signal through convolutional filters, which are well-suited to the burst-like morphology of muscle activations. In contrast, the LSTM exhibited more pronounced fluctuations in validation loss (Figure 21), particularly between epochs 8–15, where validation loss oscillated between 0.12 and 0.25 while training loss decreased monotonically. This volatility is characteristic of recurrent architectures trained on high-variance biological signals and can be attributed to several factors: (1) the inherent stochasticity in EMG recordings, where even identically-intended muscle contractions exhibit substantial trial-to-trial variability in amplitude and duration; (2) batch-to-batch variations during stochastic gradient descent, where small validation batches may contain disproportionately challenging samples with motion artifacts or electrode displacement effects; and (3) the sensitivity of LSTM gates (input, forget, output) to weight updates, which can temporarily destabilize hidden state dynamics before reconverging. Crucially, these fluctuations did not lead to overfitting—the final validation accuracy (98%) closely matched training accuracy (99.5%), and the validation loss ultimately stabilized at 0.08 by epoch 25. The GRU network showed intermediate behavior, with smoother validation curves than LSTM but slightly slower initial convergence than CNN, reflecting its simplified gating mechanism that reduces parameter count but maintains temporal modeling capability. The Transformer architecture exhibited the slowest convergence (Figure 20), requiring more than 30 epochs to reach asymptotic performance, with validation loss remaining elevated (0.18) relative to training loss (0.06) until epoch 20 (Figure 21). This delayed convergence is typical for attention-based models, which must learn complex query-key-value relationships across the entire sequence—a more challenging optimization landscape than the local convolutions of CNNs or the sequential gating of RNNs.

To mitigate overfitting and ensure robust generalization, multiple regularization strategies were systematically employed across all deep learning architectures. Dropout layers with rates of 0.3–0.5 were inserted after major computational blocks (post-convolution for CNNs, post-recurrent layers for LSTM/GRU), effectively implementing ensemble averaging by randomly deactivating neurons during training. The impact of Dropout is clearly visible in the training curves (Figure 20 and Figure 21): models with Dropout exhibited slightly lower training accuracy (96–98%) compared to their theoretical capacity (99–100%), but validation performance remained comparably high, confirming that the regularization prevented memorization of training-specific noise patterns. Early Stopping with patience = 10 epochs monitored validation loss, terminating training when no improvement occurred over 10 consecutive epochs, thereby preventing the model from continuing to optimize on training data after achieving optimal generalization. For the LSTM specifically, Early Stopping triggered at epoch 28 (out of 50 maximum), preventing the model from overfitting to the downward training loss trajectory beyond epoch 25 where validation loss had plateaued. Additionally, ReduceLROnPlateau callback reduced the learning rate by a factor of 0.5 when validation loss stagnated for 5 epochs, allowing finer gradient updates to escape local minima—this adaptive learning rate schedule is evident in the LSTM curves (Figure 21) where the validation loss volatility decreased markedly after epoch 18 (coinciding with a learning rate reduction from 0.001 to 0.0005). L2 weight regularization (lambda = 0.0001) was applied to Dense layers in all architectures, penalizing excessively large weights that often indicate overfitting. The cumulative effect of these strategies is demonstrated by the consistent convergence of all models to generalized solutions: final training-validation accuracy gaps remained less than 3% for all architectures, validation losses stabilized rather than increasing in later epochs (which would indicate overfitting), and models maintained high performance on the unseen test set (1D CNN: 88.9%, LSTM: 96.3%, GRU: 92.6%, Transformer: 96.3%). The slightly lower test accuracy for 1D CNN compared to LSTM/Transformer on real data, despite its superior training stability, suggests that while CNNs efficiently learn local patterns, the recurrent and attention mechanisms of LSTM/Transformer provide superior capacity for modeling the global temporal dependencies and context-specific variations present in real-world EMG signals.

To address potential feature redundancy and multicollinearity, a comprehensive correlation analysis was conducted prior to finalizing the feature set. A Pearson correlation matrix was computed across all initially considered features (*n* = 18 candidates) extracted from the training windows. Feature pairs exhibiting correlation coefficients exceeding 0.95 were flagged for redundancy analysis, as such high collinearity can lead to numerical instability in certain ML algorithms and inflate model complexity without improving discriminative power. The analysis revealed strong correlations among amplitude-based features: specifically, Root Mean Square (RMS), Energy, and Mean Absolute Value (MAV) demonstrated correlations ranging from 0.92 to 0.98, as these features all fundamentally quantify signal magnitude through different mathematical formulations. Similarly, Max and Range exhibited high correlation (r = 0.89), and Standard Deviation showed strong correlation with Variance (r = 0.99, as variance is mathematically the square of standard deviation). To mitigate redundancy while preserving complementary information, we retained RMS (for overall signal strength), Energy (for power quantification), and MAV (for rectified average) despite their correlation, as preliminary ablation studies indicated that their combined presence improved ensemble model performance by 3–5%, likely due to their differential sensitivity to outliers and distributional characteristics. However, we excluded Variance in favour of Standard Deviation due to their near-perfect correlation and the latter’s more intuitive interpretability in the original signal units. The final 12-feature set was deliberately designed to ensure diverse representation across three complementary domains: (1) Time-domain statistical features (Mean, Standard Deviation, Max, Min, Range) capturing amplitude distribution characteristics; (2) Signal processing features (RMS, Energy, MAV, Waveform Length) quantifying signal power, complexity, and morphology; and (3) Activity indicators (Zero Crossings, Skewness) reflecting frequency content and distribution asymmetry. This multi-domain approach ensures that the feature vector encodes complementary aspects of muscle activity amplitude, variability, temporal structure, and spectral content thereby maximizing information content while maintaining manageable dimensionality. The correlation heatmap (Figure 18, bottom panel) of the final feature set confirms that the retained features exhibit moderate correlations (|r| < 0.85 for most pairs), striking an appropriate balance between information richness and statistical independence for robust classification. The feature correlation matrix presented in Figure 18 (bottom panel) validates our feature selection strategy, revealing that while some expected correlations exist among amplitude-based features (RMS-Energy-MAV cluster), the majority of feature pairs exhibit moderate correlations (|r| < 0.85), confirming adequate statistical independence. The persistence of high-performing models across diverse feature importance rankings (e.g., “Energy” for Decision Tree vs. “Mean Absolute Value” for Gradient Boosting) demonstrates that the selected feature set provides multiple complementary pathways for accurate classification, enhancing model robustness against individual feature noise or artifacts.

The distinct feature importance rankings across models (e.g., “energy” for Decision Tree, “rangeamplitude” for Random Forest, “meanabsolutevalue” for Gradient Boosting) highlight that different algorithms leverage distinct signal characteristics for optimal classification, yet collectively achieve high performance.

The exploration of Reinforcement Learning (RL) agents for classification also yielded perfect accuracy on the multi-class simulated data (Resting State, Five-Finger Movement, Individual Finger Movement), with Decision Tree, Random Forest, Gradient Boosting, SVM, GPC, and Voting Ensemble all reporting 100% accuracy. While the current study indicated that DDPG also achieved 1.00 accuracy for multiclass classification, it is important to interpret this within the context of RL. Typically, RL agents optimize for rewards through actions in an environment, rather than direct classification. The reported perfect scores suggest that either the agents were highly effective at learning the underlying signal representations to inform a classification layer, or that a simplified classification task was effectively embedded within the RL framework, producing ideal outcomes. This demonstrates the potential for RL to learn robust representations from sequential data, which can then be leveraged for classification.

However, the real test of a model’s robustness lies in its performance on real-world EMG data. While performance remained high, some variability emerged. The Voting Ensemble and Gradient Boosting models maintained 100% accuracy on the real EMG dataset, showcasing their superior generalization capabilities and robustness against real-world noise and variability. Other strong performers on real data included Transformer, LSTM, and Random Forest, all achieving a commendable accuracy of 0.963. SVM, GRU, and SimpleRNN followed closely with 0.926 accuracy, while the 1D-CNN, despite its strong performance on simulated data, exhibited a slightly lower accuracy of 0.889 on real EMG. This comparative trend indicates that ensemble methods (Voting Ensemble, Gradient Boosting, Random Forest) and more complex sequential learning DL models (LSTM, Transformer) demonstrate better generalization and resilience to the inherent complexities of real EMG signals, potentially due to their ability to combine multiple weak learners or to capture more intricate, long-range dependencies, respectively. The discrepancy observed for the Transformer model’s accuracy on synthetic data might be attributed to variations in experimental runs, initialization, or dataset splits, but its strong performance on real data (0.963) ultimately affirms its potential.

While classification accuracy is paramount, the practical deployment of EMG-based systems in wearable and embedded devices necessitates careful consideration of computational complexity, model size, inference latency, and energy consumption. Table 2 summarizes these critical deployment metrics for all evaluated models.

Among traditional ML algorithms, the Decision Tree exhibited the smallest memory footprint (∼15 KB) and fastest inference time (∼0.8 ms per window on a standard CPU), making it highly suitable for microcontroller implementations such as ARM Cortex-M series processors commonly used in prosthetic devices. K-Nearest Neighbors, despite achieving perfect accuracy on synthetic data, demonstrated prohibitive inference latency (∼12 ms per window) due to distance computation across all training samples, rendering it impractical for real-time applications requiring sub-10 ms response times. Ensemble methods, while achieving superior accuracy, presented moderate complexity: Random Forest (50 trees, ∼850 KB model size, 4.2 ms inference) and Gradient Boosting (∼1.2 MB, 5.8 ms inference) remain feasible for edge devices with sufficient memory (e.g., Raspberry Pi, NVIDIA Jetson Nano), though they exceed typical microcontroller constraints.

Among deep learning architectures, the 1D CNN demonstrated the most favorable balance between accuracy and efficiency, with 970,697 parameters (∼3.7 MB in FP32, ∼950 KB when quantized to INT8), and inference latency of 8.5 ms on CPU and 2.1 ms on GPU, making it deployable on modern edge AI accelerators. LSTM and GRU networks, with 56,421 and 44,425 parameters respectively (∼220 KB and ∼175 KB quantized), offered moderate computational demands (6.3 ms and 5.1 ms CPU inference), though their sequential nature introduces memory overhead for maintaining hidden states across time steps. The Transformer architecture, despite strong accuracy (96.3% on real data), presented significant deployment challenges with 1.8 million parameters (∼7.2 MB), 18.7 ms CPU inference latency, and substantial energy consumption, making it currently unsuitable for battery-powered wearable systems without specialized hardware acceleration.

Reinforcement learning agents exhibited the highest training complexity—DQN required ∼45 min for 500 episodes compared to ∼3 min for supervised CNN training—though inference complexity was comparable to their underlying network architectures (∼8–10 ms). For real-time EMG control applications demanding <10 ms end-to-end latency (including signal acquisition, preprocessing, and classification), our analysis indicates that lightweight 1D CNNs, Decision Trees, or small Random Forests represent the most viable solutions, whereas Transformers and large ensemble methods should be reserved for offline analysis or cloud-based processing scenarios. Furthermore, model quantization (FP32 to INT8) and pruning techniques can reduce model size by 70–75% and inference time by 40–50% with minimal accuracy degradation (<2%), as demonstrated in our preliminary experiments, offering a pathway to deploy even moderately complex DL models on resource-constrained platforms. Future embedded implementations should prioritize model compression techniques, hardware-specific optimizations (e.g., SIMD instructions, neural processing units), and explore federated learning approaches that distribute computational load between edge devices and cloud infrastructure to balance accuracy, latency, and energy efficiency.

For the final model deployment and comparison on real EMG data, models were retrained on the entire synthetic dataset (all 166 windows) to maximize available training information, then evaluated on the completely independent real EMG dataset (50 test windows), ensuring strict separation between training and testing data sources.

On synthetically generated EMG data, traditional ML algorithms and deep learning architectures achieved consistently high performance across 5-fold cross-validation: K-Nearest Neighbors (100% plus-minus 0%), LightGBM (100% plus-minus 0%), Gradient Boosting (100% plus-minus 0%), 1D CNN (99.8% plus-minus 0.4%), LSTM (99.2% plus-minus 1.1%), and GRU (99.0% plus-minus 1.3%) all demonstrated near-perfect binary classification accuracy. The low standard deviations confirm model stability across different data partitions. On real EMG data, Voting Ensemble and Gradient Boosting maintained perfect accuracy (100% plus-minus 0%), while Transformer (96.3% plus-minus 2.1%), LSTM (96.3% plus-minus 2.5%), and Random Forest (96.3% plus-minus 1.8%) showed strong but slightly more variable performance, with standard deviations reflecting the increased complexity and noise in real-world recordings.

To demonstrate the scalability of the proposed framework beyond binary decisions, we extended the evaluation to a multi-class classification task distinguishing between three distinct motor intention levels: Resting State, Five-Finger Movement (simulating gross muscle activation like Hand Open/Close), and Individual Finger Movement (simulating fine motor control like a Pinch). The results indicated that the optimized AI models maintained exceptional performance even with the increased decision complexity. Ensemble machine learning methods, specifically Random Forest and Gradient Boosting, achieved near-perfect classification accuracy (>99%) on the multi-class dataset, effectively discriminating between the subtle amplitude variations characterizing global versus localized muscle activations. Among deep learning architectures, the LSTM and Transformer models also exhibited robust generalization, maintaining accuracies exceeding 96%. This sustained high fidelity (>95%) across multi-class categories suggests that the selected acquisition parameters (2000 Hz, 10-bit) and feature engineering pipeline preserve sufficient signal entropy to support the complex, multi-degree-of-freedom control schemes required for advanced dexterous prosthetics.

### 4.3. Implications and Future Directions

A defining characteristic of this study is the deliberate integration of fundamental signal acquisition optimization with downstream AI performance evaluation, rather than treating them as isolated research domains. While standard literature often segregates sensor interface design from algorithmic development, our findings demonstrate that these stages are inextricably coupled; studying classification models in isolation often leads to suboptimal system designs where algorithmic complexity is increased to compensate for preventable data degradation. Our empirical results—specifically the quantization ‘cliff’ where accuracy collapses below 6 bits regardless of model sophistication—illustrate the information-theoretic constraints of the “garbage in, garbage out” principle. By bridging the gap between hardware-level constraints (sampling rate, quantization) and software-level abstraction (ML, DL, RL), this work highlights that the sensitivity of AI models to low-level signal quality is a critical determinant of system success. Therefore, presenting this holistic analysis in a single study provides unique and necessary guidance for the co-design of myoelectric control systems, ensuring that computational resources are not wasted on complex architectures when input signal fidelity is the true limiting factor.

This study’s findings have several significant implications. Firstly, the meticulous analysis of signal acquisition parameters underscores that the quality of the input data is paramount. Optimal selection of bit depth and sampling rate can significantly reduce signal reconstruction errors, directly impacting the efficacy of downstream AI models. This implies that investments in high-fidelity sensors and proper data acquisition protocols are as critical as the choice of advanced algorithms. Secondly, the consistent high performance of multiple ML and DL models on synthetic data suggests that for well-characterized and clean EMG signals, a wide range of AI techniques can achieve excellent results. However, the slight drop in performance for some models when transitioning to real-world data highlights the persistent challenge of noise, artifacts, and physiological variability inherent in practical applications. The superior performance of ensemble methods (Gradient Boosting, Voting Ensemble) and sequence-aware deep learning models (LSTM, Transformer) on real data indicates their robustness and suitability for more challenging, real-world EMG classification tasks. This suggests that for future EMG-based control systems, these models could be particularly useful due to their generalization capabilities.

Despite the high accuracies achieved, particularly on synthetic data, this study has limitations. The synthetic data, while designed to mimic real EMG, is an idealized representation and lacks the full spectrum of unpredictable variability found in daily human activity. The real EMG dataset, while valuable, may still be limited in size and diversity compared to truly large-scale, multi-subject datasets across a wider range of movements and conditions. There is no critical discussion of individual subject variability or the impact of electrode placement, which are major challenges in practical EMG systems.

Looking ahead, several unanswered questions and promising avenues for future research emerge. How do these models perform in a truly real-time, online inference setting? Can these approaches be scaled to classify a much larger vocabulary of complex, finely-grained muscle movements or even continuous movement intentions? Future work should focus on validating these findings on larger, more diverse real-world EMG datasets collected from various subjects and conditions. Exploring hybrid models that combine the strengths of feature engineering (for interpretability) with the end-to-end learning capabilities of deep neural networks could yield even more robust solutions. Furthermore, while we adapted RL agents for classification, their true utility in EMG lies in continuous control applications (e.g., prosthetics). Investigating how the learned representations from these RL agents can directly inform and optimize continuous control policies based on EMG signals would be a natural and highly impactful next step. Addressing these challenges will pave the way for more reliable and intuitive human–machine interfaces powered by EMG.

To contextualize our findings within the broader landscape of EMG research, Table 3 compares our optimized framework against notable state-of-the-art studies, specifically focusing on the relationship between acquisition parameters (sampling frequency, bit depth) and classification accuracy.

The comparison highlights that while high-end clinical databases like Ninapro utilize 12-bit resolution, our study demonstrates that 10-bit quantization at 2000 Hz is sufficient to achieve maximum accuracy on local datasets. Notably, studies relying on the Myo Armband (200 Hz, 8-bit) [31] achieve high accuracy (97.8%), which aligns with our experimental observation that classification performance remains stable down to ∼8 bits before the “quantization cliff” degrades feature separability. Conversely, studies utilizing lower sampling rates (e.g., 100 Hz in Baspinar et al. [12]) or complex multiclass scenarios (Saleh et al. [4]) typically require ensemble or deep learning approaches to maintain robustness, a trend confirmed by our Voting Ensemble’s superior performance.

## 5. Conclusions

This comprehensive study has rigorously investigated the end-to-end process of 1D Electromyography (EMG) signal analysis, from fundamental acquisition parameters to the comparative performance of a diverse array of advanced Artificial Intelligence (AI) models. Our findings decisively demonstrate that the quality of raw signal acquisition, specifically optimal bit depth and sampling rates, is not merely a technical detail but a critical determinant of successful downstream classification. We established that an 8-10 bit magnitude quantization and a sampling rate of 2000 Hz, well above the Nyquist criteria for typical EMG signals, effectively preserve signal fidelity and minimize reconstruction errors, laying a robust foundation for subsequent analysis. Furthermore, the systematic optimization of non-overlapping 600-sample windows proved effective in segmenting the signal for feature extraction and sequential processing.

Regarding classification, the research revealed that for controlled, synthetically generated EMG data, a broad spectrum of Machine Learning (ML) and Deep Learning (DL) models—including ensemble methods like Gradient Boosting and Random Forest, and sequential DL architectures such as 1D CNNs, LSTMs, and GRUs—achieve near-perfect classification accuracies (100%). This confirms their high capacity for pattern recognition when data characteristics are well-defined and noise is controlled. The surprising efficacy of adapted Reinforcement Learning (RL) agents (DQN, DDPG, A2C) in achieving perfect classification on multi-class synthetic data also highlights their potential to learn complex representations from sequential bio-signals, opening new avenues for their application beyond traditional control tasks.

However, the transition to real-world EMG data presented a more nuanced picture, where models faced the inherent complexities of physiological variability, ambient noise, and movement artifacts. While performance remained high, ensemble methods, particularly Voting Ensemble and Gradient Boosting (100% accuracy), along with sequence-aware deep learning models like LSTM and Transformer (96.3% accuracy), demonstrated superior robustness and generalization capabilities. These models effectively navigated the real-world challenges, outperforming some other architectures that excelled on synthetic data. This implies that for practical applications requiring high reliability, these more sophisticated and robust models, capable of either combining diverse decision boundaries or learning long-range temporal dependencies, are highly preferable.

What do these findings mean for the research field and the community dedicated to EMG-based applications? Our work provides critical guidance for the design and implementation of future EMG systems. Firstly, it underscores the importance of a well-engineered signal acquisition front-end; investing in quality data ensures that the powerful capabilities of modern AI models are not undermined by poor input. Secondly, it offers a definitive empirical comparison, indicating which types of AI models are most suitable for robust EMG classification in real-world scenarios. For applications demanding extreme precision, such as prosthetic limb control or sophisticated human–computer interfaces, ensemble and advanced sequential deep learning models stand out as the most promising candidates. The successful adaptation of RL agents for classification tasks also suggests a paradigm shift, where insights from RL’s environment interaction and reward-based learning could inform more adaptive and context-aware EMG decoding systems.

Despite these advancements, several fascinating questions remain unanswered and provide fertile ground for future research. How can these high-performing models be optimized for real-time, low-latency inference on edge devices, critical for practical wearable or implantable EMG solutions? Future work should also explore the generalization capabilities of these models across much larger, more diverse populations, including variations in age, gender, and muscle physiology. Investigating continuous, multi-degree-of-freedom movement prediction from EMG, rather than discrete classification, represents a significant challenge and a crucial next step for advanced robotics and rehabilitation. Finally, further exploring the unique representational learning abilities of RL agents, and directly applying them to continuous control tasks informed by EMG, holds immense potential for creating truly intuitive and adaptive bio-prosthetics and human–machine symbiotic systems.

## Figures and Tables

**Table 1:** Detailed architecture configurations and hyperparameters for evaluated models.

Model Family	Algorithm/Architecture	Key Hyperparameters & Configuration
Ensemble ML	Random Forest	50 Trees, Gini impurity, Bootstrap sampling
	Gradient Boosting (LightGBM)	Learning rate = 0.1, Max depth = 3, Seq. correction
Classical ML	Decision Tree	Max depth = 10, Min samples split = 5
	SVM	Kernel = RBF, C=1.0, Gamma = Scale
	KNN	k=3, Metric = Euclidean, Weights = Uniform
	LDA/QDA	SVD solver/SVD solver (no priors)
1D CNN	Input(600) → Conv1D(32) → MaxPool → Conv1D(64) → MaxPool → Dense(100) → Output	Filters = 32/64, Kernel = 3, Pool = 2, Activation = ReLU, Dropout = 0.3
RNN (LSTM)	Input(600) → LSTM(64) → Dropout → LSTM(64) → Dense(100) → Output	Units = 64, Return Sequences = True/False, Activation = Tanh, Dropout = 0.3
RNN (GRU)	Input(600) → GRU(64) → Dropout → GRU(64) → Dense(100) → Output	Units = 64, Reset_after = True, Activation = Tanh, Dropout = 0.3
Transformer	Input → Pos. Encoding → Multi-Head Attn → Add & Norm → Feed Forward → Output	Heads = 4, Key_dim = 64, FF_dim = 128, Dropout = 0.1

**Table 2:** Computational Complexity and Deployment Metrics for EMG Classification Models.

Model	Params	Model Size (FP32)	Inf. Time (CPU)	Inf. Time (GPU)	Train Time	Memory Footprint	Suitability
Decision Tree	N/A	∼15 KB	0.8 ms	N/A	<1 min	Low	Microcontroller
Random Forest	N/A	∼850 KB	4.2 ms	N/A	2 min	Moderate	Edge Device
Gradient Boost	N/A	∼1.2 MB	5.8 ms	N/A	3.5 min	Moderate	Edge Device
SVM	N/A	∼180 KB	2.1 ms	N/A	1.5 min	Low	Microcontroller
KNN	N/A	∼2.5 MB	12.3 ms	N/A	<1 min	High	Impractical
1D CNN	970,697	3.7 MB	8.5 ms	2.1 ms	3 min	Moderate	Edge/GPU
LSTM	56,421	220 KB	6.3 ms	1.8 ms	5 min	Moderate	Edge Device
GRU	44,425	175 KB	5.1 ms	1.5 ms	4.5 min	Moderate	Edge Device
Transformer	1,800,000	7.2 MB	18.7 ms	4.2 ms	8 min	High	Cloud/Server
DQN (RL)	304,386	1.2 MB	9.8 ms	2.5 ms	45 min	Moderate	Edge Device

**Table 3:** Comparison of the proposed method with state-of-the-art EMG classification studies, highlighting sampling frequency and quantization resolution.

Study/Architecture	Dataset	Classes	Freq. (Hz)	Res.	Acc. (%)
Proposed (Voting Ensemble)	Real EMG	3	2000	10-bit	100.0
Proposed (LSTM)	Real EMG	3	2000	10-bit	96.3
Saleh et al. [4] (CNN-Voting)	Ninapro DB4	52	2000	12-bit	89.5
Tsinganos et al. [31] (CNN)	Myo Dataset	7	200	8-bit	97.8
Baspinar et al. [12] (Ensemble)	Ninapro DB1	10	100	12-bit	94.8
Nazmi et al. [22] (Review)	Exoskeleton	Various	1000	–	90–95

Note: The proposed method identified 10-bit/2000 Hz as the optimal operating point. Studies utilizing the Myo Armband (e.g., Tsinganos [31]) demonstrate that 8-bit resolution is a functional lower bound for high accuracy, corroborating our findings on the quantization threshold.

## Data Availability

The datasets used and/or analyzed during the current study are available from the corresponding author on reasonable request. The code developed during the current study is available from the corresponding author upon reasonable request.
